# Acute Colonic Pseudo-Obstruction After Ventriculoperitoneal Shunt Placement for Normal Pressure Hydrocephalus

**DOI:** 10.7759/cureus.8295

**Published:** 2020-05-26

**Authors:** Cherry Liu, Daniel Smerin, Isin Comba, Lakhinder Bhatia

**Affiliations:** 1 Medicine, University of Central Florida College of Medicine, Orlando, USA; 2 Internal Medicine, University of Central Florida College of Medicine, Orlando, USA

**Keywords:** ventriculoperitoneal shunt, ogilvie's syndrome, colonic pseudo-obstruction

## Abstract

Ogilvie’s syndrome is a rare postoperative condition commonly referred to as a “colonic pseudo-obstruction” due to the absence of mechanical obstruction. It should be a differential for patients over the age of 60 years who present with nausea, vomiting, and colonic dilatations on imaging. Ogilvie’s syndrome following a ventriculoperitoneal (VP) shunt placement is an extremely rare entity with only one other adult patient reported in the English literature. In this case report, we explore the diagnosis and management of a 76-year-old patient who presented with abdominal pain and multiple bouts of bilious, non-bloody vomitus two days after a ventriculoperitoneal shunt. The ultimate diagnosis of Ogilvie's syndrome along with imaging and subsequent management is detailed, and diagnosis guidelines and treatment options for Ogilvie's syndrome are analyzed and explained. This case highlights the importance of keeping Ogilvie's syndrome on the list of differentials in a postoperative patient in all abdominal surgeries, even if they are minimally invasive.

## Introduction

Ogilvie’s syndrome is defined as an acute dilatation of the colon in the absence of mechanical obstruction. It is a relatively rare clinical entity with an estimated annual incidence about 100 cases per 100,000 hospital admissions and more common in men over the age of 60 years in the United States [1]. A postoperative state poses significant risk for Ogilvie's syndrome, as 40%-50% of documented cases of acute colonic pseudo-obstruction occur after surgery or trauma, most commonly after coronary artery bypass grafting [2]. In this report, we present a 76-year-old man who developed Ogilvie’s syndrome on postoperative day 2 after a ventriculoperitoneal (VP) shunt placement. While there can be intra-abdominal complications secondary to VP shunting, more complications are shunt infections followed by cerebral spinal fluid pseudocyst, abscess, and infected fluid [3]. There is only one adult case of Ogilvie’s syndrome following VP shunt surgery for normal pressure hydrocephalus in the English literature [4].

## Case presentation

**Figure 1:**
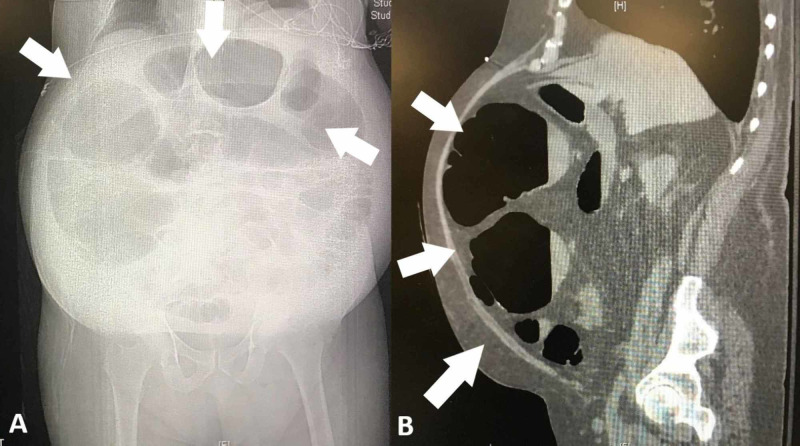
Abdominal X-ray revealing moderate to severe dilatation of the colon (A). CT scan with oral contrast showing a severe dilatation of colon (B).

A 76-year-old man with a past medical history of seizures, NPH, and obstructive sleep apnea was admitted to the hospital for an elective VP shunt for NPH. His past surgeries included laparoscopic cholecystectomy, open appendectomy, and two umbilical hernias including one with a mesh instalment. The surgery went smoothly with no immediate complications and the patient recovered well until on postoperative day 2; he developed abdominal distention, constant lower quadrant pain, and multiple bouts of bilious, non-bloody vomitus. His physical exam included vital signs within normal ranges, and was significant for a distended, tympanic abdomen with generalized tenderness, but no rebound or guarding. Initial laboratory tests (complete blood count and complete metabolic panel) were unremarkable. An X ray (Figure 1A) and computed tomographic (CT) scan (Figure 1B) of the abdomen with oral contrast showed severe dilatation of the right and transverse colon with a relative transition section at the splenic flexure. The small bowel was noted to be normal in diameter with no evident obstruction.

**Figure 2:**
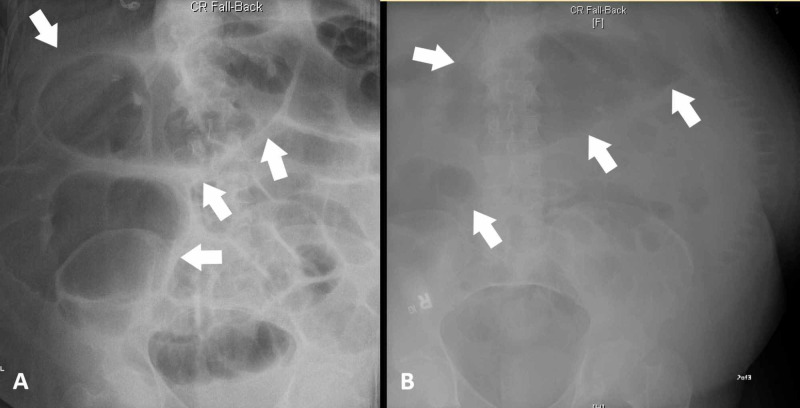
(A) Abdominal X-ray in the supine view acquired before the colonoscopy revealing multiple dilated bowel loops that are seen throughout the abdomen. (B) Abdominal X-ray acquired after the decompressive colonoscopy showing some air-filled loops less pronounced than before.

Gas and stool were noted in the rectum. The VP shunt was visualized terminating in the right upper abdominal quadrant around the subhepatic region, and a VP series was not indicated. A nasogastric (NG) tube was placed, and the patient was closely followed with serial abdominal exams and X-rays. On postoperative day 3, he was feeling better in terms of abdominal distension and pain with an NG tube in place, but still failed to have any flatus or bowel movements. Thus, he was started on docusate taken orally, and given a fleet enema. Finally, on postoperative day 5, the patient had a bowel movement and flatus, which significantly reduced his abdominal pain and resolved his nausea and vomiting. However, on day 8, his abdominal pain and distension returned. Due to recurrence of the abdominal pain and distension on conservative management, on postoperative day 8, the patient underwent a decompressive colonoscopy that resulted in the improvement of his abdominal distention, cramping, and pain. A fecal tube was placed following the procedure. However, the symptoms of abdominal distension and pain returned, and he underwent another decompressive colonoscopy, which revealed minimal peristalsis in the examined colonic sections. Abdominal X-rays obtained before (Figure 2A) and after (Figure 2B) the second decompressive colonoscopy showed some improvement in colonic distension. 

The patient had a prolonged hospital stay due to persistent recurrent symptoms of nausea, vomiting, abdominal distension, and pain. He responded to the conservative management, did not require another colonoscopy, and was discharged from the hospital after the complete resolution of his symptoms.

## Discussion

In 1948, Ogilvie’s syndrome was first described by Sir William Ogilvie, who noted two patients with massive colonic dilations that occurred without mechanical obstruction [1]. This type of colonic dilation is now referred to as a “pseudo-obstruction”. At the time, Sir William Ogilvie suspected that this pseudo-obstruction was due to neurological derangements that are often seen in malignant disease spreading to the nervous supply [1]. However, more recently the new theory is that there is large-bowel parasympathetic dysfunction. This theory has been supported by the use of parasympathomimetic agents for complete resolution [5]. A postoperative state poses a significant risk factor for Ogilvie's syndrome, as many of the cases in literature review of acute colonic pseudo-obstructions occur after surgery or trauma [6]. While elderly patients with various comorbid conditions are disproportionately affected, Ogilvie’s syndrome can be a sequelae of general surgical procedures [7]. Table 1 outlines the results of two large studies including more than 1,000 cases of Ogilvie’s syndrome and the known associated conditions of the patients [6,8]. Among the postoperative conditions, neurosurgery ranked at the lowest rate at 1% and the cause is idiopathic [6]. 

**Table 1:** Underlying Conditions in 1,211 Patients From Two Major Studies on Ogilvie’s Syndrome

Condition	Example	Number	Percentage
Postoperative conditions	Coronary bypass, orthopedic surgery (hip especially), urology surgery (including kidney transplants), thoracic and cardiovascular surgery, neurosurgery	305	25.2%
Non-operative trauma	Fractures, burns	138	11.4%
Pregnancy/gynecologic issues	Cesarean section, gynecological issues, normal pregnancy, vaginal delivery	76	6.3%
Cardiopulmonary disorders	Myocardial infarction, congestive heart failure, chronic obstructive pulmonary disease, acute respiratory failure	208	17.2%
Neurologic disorders	Dementia, Parkinson’s disease, stroke, Alzheimer’s disease, spinal cord injury	104	8.6%
Cancer	Malignant diseases, chemotherapy	69	5.7%
Intra-abdominal disorders	Hepatic, gastrointestinal, pancreatitis, alcoholism (ascites), cholecystitis	70	5.8%
Systemic disorders	Endocrine and metabolic disease, infection, sepsis, intoxication	190	15.7%
Retroperitoneal disorders	Acute renal failure, kidney disease	49	4.0%
Idiopathic	Unknown	58	4.7%

Ogilvie’s syndrome is considered a diagnosis of exclusion. Patients commonly present with abdominal distension, discomfort, nausea, and vomiting. Interestingly, some patients will even retain some degree of bowel function resulting in diarrhea due to water hypersecretion. Diagnosis typically requires an abdominal imaging, with CT scan with oral contrast as the gold standard of diagnosis. It is important to utilize the CT imaging to rule out perforation and anatomic or mechanical obstruction. If such imaging is not available, a contrast enema study has been shown to have a sensitivity of 96% for Ogilvie’s syndrome. Most importantly, diagnostic colonoscopy should not be performed due to increased risk of perforation [1].

Treatment for Ogilvie’s syndrome involves conservative, pharmacological, and surgical management. In order to pursue this avenue of treatment, it is important to first rule out mechanical obstruction, ischemia, and perforation [9]. The first-line treatment for Ogilvie’s syndrome is conservative management [1]. This consists of observation [6]. In patients with mild uncomplicated Ogilvie’s syndrome, initial management includes keeping the patient "nothing by mouth" in addition to the placement of an NG tube to help with decompression [1]. Follow-up imaging should be utilized to determine the efficacy of treatment [1]. Ultimately, this conservative treatment should be used for up to 72 hours; however, if there is no improvement, pharmacologic therapy should be initiated with agents like acetylcholinesterase inhibitors (e.g. neostigmine) that enhance bowel movements [9]. Colonic decompression through colonoscopy is used when conservative and pharmacologic management is insufficient to resolve the pseudo-obstruction [6]. Surgical management is reserved for patients who develop complications such as ischemia or bowel perforation [1]. The efficacy of surgery has been reported to be 50% alone, but up to 88% if there was concurrent decompression tube placement [2]. Many patients also require multiple decompressions [10].

## Conclusions

Ogilvie’s syndrome following a VP shunt placement is an extremely rare complication and can prolong the hospital course. Thus, it is important to have this condition considered as a differential for postoperative patients who experience abdominal pain and discomfort following a VP shunt placement. While most intra-abdominal complications following this surgery are infectious in nature, close follow-up of patients experiencing abdominal discomfort along with lack of bowel movement is important, as Ogilvie’s syndrome can ultimately lead to serious complications such as ischemia and bowel perforation if unrecognized. 
